# Angiogenic content of microparticles in patients with diabetes and coronary artery disease predicts networks of endothelial dysfunction

**DOI:** 10.1186/s12933-022-01449-0

**Published:** 2022-02-02

**Authors:** Isra Marei, Omar Chidiac, Binitha Thomas, Jennifer Pasquier, Soha Dargham, Amal Robay, Muneera Vakayil, Mohammad Jameesh, Christopher Triggle, Arash Rafii, Amin Jayyousi, Jassim Al Suwaidi, Charbel Abi Khalil

**Affiliations:** 1 Department of Pharmacology, Weill Cornell Medicine-Qatar, Doha, Qatar; 2 National Heart and Lung Institute, Imperial College London, London, UK; 3 Department of Genetic Medicine, Weill Cornell Medicine-Qatar, Doha, Qatar; 4 Biostatistics Core, Weill Cornell Medicine-Qatar, Doha, Qatar; 5 Heart Hospital, Hamad Medical Corporation, Doha, Qatar; 6 Department of Endocrinology, Hamad Medical Corporation, Doha, Qatar; 7 Joan and Sanford I. Weill Department of Medicine, Weill Cornell Medicine, New York, USA

**Keywords:** Diabetes, Microparticles, Coronary artery disease, Acute coronary syndrome, Endothelial dysfunction, Cardiovascular disease

## Abstract

**Background:**

Elevated endothelial microparticles (EMPs) levels are surrogate markers of vascular dysfunction. We analyzed EMPs with apoptotic characteristics and assessed the angiogenic contents of microparticles in the blood of patients with type 2 diabetes (T2D) according to the presence of coronary artery disease (CAD).

**Methods:**

A total of 80 participants were recruited and equally classified as (1) healthy without T2D, (2) T2D without cardiovascular complications, (3) T2D and chronic coronary artery disease (CAD), and (4) T2D and acute coronary syndrome (ACS). MPs were isolated from the peripheral circulation, and EMPs were characterized using flow cytometry of CD42 and CD31. CD62E was used to determine EMPs’ apoptotic/activation state. MPs content was extracted and profiled using an angiogenesis array.

**Results:**

Levels of CD42- CD31 + EMPs were significantly increased in T2D with ACS (257.5 ± 35.58) when compared to healthy subjects (105.7 ± 12.96, p < 0.01). There was no significant difference when comparing T2D with and without chronic CAD. The ratio of CD42-CD62 +/CD42-CD31 + EMPs was reduced in all T2D patients, with further reduction in ACS when compared to chronic CAD, reflecting a release by apoptotic endothelial cells. The angiogenic content of the full population of MPs was analyzed. It revealed a significant differential expression of 5 factors in patients with ACS and diabetes, including TGF-β1, PD-ECGF, platelet factor 4, serpin E1, and thrombospondin 1. Ingenuity Pathway Analysis revealed that those five differentially expressed molecules, mainly TGF-β1, inhibit key pathways involved in normal endothelial function. Further comparison of the three diabetes groups to healthy controls and diabetes without cardiovascular disease to diabetes with CAD identified networks that inhibit normal endothelial cell function. Interestingly, DDP-IV was the only differentially expressed protein between chronic CAD and ACS in patients with diabetes.

**Conclusion:**

Our data showed that the release of apoptosis-induced EMPs is increased in diabetes, irrespective of CAD, ACS patients having the highest levels. The protein contents of MPs interact in networks that indicate vascular dysfunction.

**Supplementary Information:**

The online version contains supplementary material available at 10.1186/s12933-022-01449-0.

## Background

Cardiovascular disease (CVD) has the highest death rate in the world [1, 2]. Adults with type 2 diabetes (T2D) have a two-fold increased risk of developing cardiovascular complications and a higher death rate and morbidity than individuals without diabetes [3–5]. Furthermore, dying from CVD, including coronary artery disease (CAD), occurs in 75% of diabetic people [6].

T2D is associated with chronic low-grade inflammation and metabolic stress, leading to endothelial dysfunction and platelet hyperaggregation [7, 8]. Evidence suggests that the inflammatory pathways might be the common modulator of the pathogenesis of T2D [9]. It is suggested that such modulation occurs through the secretion of extracellular vesicles by the cells to the circulation [7]. Microparticles (MPs) and exosomes are the two cell-derived microvesicles found in human body fluids are microparticles (MPs) and exosomes. MPs are extracellular vesicles with sizes ranging between 100 and 1000 nm of diameter [10]. Various types of cells secrete these microparticles in response to chemical or physical stimuli during the apoptosis or activation states of the cells [11]. Platelets release most circulating microparticles; however, leukocytes, erythrocytes, and endothelial cells are also capable of secreting microparticles [11].

Endothelial microparticles (EMPs) circulate at low levels in the blood of healthy subjects [12–14], while there was a reported increase in EMPs levels in different CVD pathologies [15–17]. There is a need to establish the role of EMPs in developing diabetic cardiovascular complications and their link to disease prognosis/severity. Thus, in this study, we investigated the release of EMPs in the blood of diabetes patients with and without CAD. Through a flow cytometric approach, we quantified the release of EMPs in relation to the disease state. We identified variations in the percentages of activated and apoptotic EMPs in each case. Finally, we examined the content of the total circulating population of the isolated MPs and the expression of angiogenic factors.

## Methods

### Research participants

A total of 80 research participants were recruited and classified under four groups: (I) healthy participants without T2D or CVD (Controls, n = 20), (II) T2D patients without micro- or macro-vascular complications (T2D, n = 20), (III) T2D, and chronic coronary artery disease (CCAD, n = 20), and (IV) T2D and acute coronary syndrome (ACS) (ACS, n = 20). Healthy non-diabetic controls are participants with good overall health without diabetes (glucose < 5.5 mmol/L and HbA_1c_ < 5.7%) or a history of a previous cardiovascular event. All T2D patients were identified as having an HbA_1C >_ 6.5%. Patients without macrovascular disease were selected based on their medical files’ absence of macrovascular disease, a previous cardiovascular event, or micro-vascular disease (diabetic retinopathy, nephropathy, or neuropathy). ACS regrouped patients with unstable angina, ST elevation, and non-ST elevation myocardial infarction (STEMI and NSTEMI) according to the American College of Cardiology and the American Heart Association classification [18]. Chronic CAD is defined as the presence of an angiographically proven coronary atherosclerotic disease. Recruitment of research participants occurred at the Heart Hospital and the Department of Endocrinology and Diabetes clinic at Hamad Medical Corporation (HMC) in Qatar. Healthy participants serving as controls were approached and recruited from the outpatient department at HMC. After eligibility assessment, clinical data were recorded, blood was withdrawn from the forearm veins to measure biochemical parameters. Another tube was transported directly to Weill Cornell Medicine – Qatar (WCM-Q)’s lab to analyze MPs.

### MPs isolation

ExoQuick-LP™ (System Biosciences, Mountain View, CA) was used to isolate MPs following the manufacturer’s recommendations. Whole blood (10 ml) was collected from patients in heparin vacutainer tubes (BD Biosciences, NJ, USA). The sample was centrifuged, and plasma was collected and centrifuged, then platelet-free plasma aliquots (250 µl) were treated with 63 µl of ExoQuick-LP and incubated overnight at 4 ºC. The sample was spun for 30 min at 1500 *g* to isolate the MPs-rich pellet.

### Flow cytometric analysis of EMPs

To quantify EMPs, MPs were stained with the endothelial markers’ fluorescein-conjugated CD31 (CD31-FITC) and phycoerythrin-conjugated E-selectin (CD62E-PE). To exclude MPs from platelet origin, phthalocyanine-conjugated CD42b (CD42b-APC) was added to the samples. MPs were incubated with all three antibodies combined to assess a positive CD62 and CD31 and a negative CD42b. For repeatability, all MPs measurements were performed twice. Control IgG antibodies were used to optimize CD42b − CD62 + and CD42b − CD31 + microparticles levels. Size calibration was performed using polystyrene microspheres (0.2 to 10 μm). MPs of less than 1.5 μm were detected by their size and density based on forward, and side scatter plots. The assessment was performed by 2-color fluorescence histogram plots as CD42b − CD62 + and CD42b − CD31 + MPs (Additional file 1: Fig. S1. a–c). MPs were analyzed with BD LSRFortessa™ Cell Analyzer-BD biosciences. The absolute counts of the CD31 and CD62 populations were calculated using the following equation (Additional file 1: Fig. S1. d): $\frac{\mathrm{CD}31\mathrm{ or CD}62/\mathrm{ Bead Count}}{\mathrm{Bead Concentration}/\mathrm{ Total Volume}}\mathrm{ X Dilution Factor}$. The expression of CD31 (endothelial cell marker) and CD62E (E selectin expressed in endothelial cells) should show positivity in endothelial MPs (EMPs). CD42-CD62 + /CD42-CD31 + ratio was then calculated to demonstrate whether the EMPs are produced by apoptotic or activated endothelial cells [16, 19, 20]. All used antibodies and reagents were purchased from BD Biosciences, NJ, USA.

### Extraction of proteins from MPs

MPs pellets were treated with RIPA lysis buffer (Sigma, MO, USA) and protease and phosphatase inhibitor cocktail (Thermo Scientific, IL, USA), followed by centrifugation for 10 min at 4 ºC. The supernatant containing the protein was collected and quantified using Bradford assay (Biorad). Optical density (OD) was measured at a wavelength of 595 nm using a plate reader (ClarioStar, Aylesbury, UK).

### Angiogenesis profiling

Relative expression of 55 angiogenesis-related proteins was determined using the Human Angiogenesis Proteome Profiler™ Array kit (R&D Systems, Abingdon, UK) following the manufacturer’s instructions as previously reported [21]. The method is detailed in the Additional file 1.

### Ingenuity Pathway Analysis (IPA)

The differentially expressed proteins from the angiogenesis profiler assay were further analyzed using IPA software to identify the top affected pathways and how these pathways are predicted to affect endothelial cell function. Networks were built using Ingenuity® Pathway analysis (IPA) software (Qiagen).

### Western blot

Western blot analysis was carried out as previously described [22]. A list of the used antibodies is detailed in supplementary data. SuperSignal™ West Dura extended duration substrate (Thermo scientific #PI34075) was used to develop the blots. Data were collected from ChemiDoc™ Imaging System (Biorad) and analyzed using Image Lab software.

### Statistical analysis

Data are represented as mean ± standard error of the mean (SEM), mean with standard deviation (SD), or median with interquartile range (IQR) as appropriate. Data trends were visually and statistically evaluated for normality. Correlation between EMPs and patients’ characteristics was done using the non-parametric Spearman correlation test. Multiple linear regression analysis was performed with EMPs ratio as the dependent variable, and each of the baseline characteristics of the studied groups as the independent variable (including BMI, diastolic blood pressure, systolic blood pressure, total cholesterol, triglycerides, LDL-C, and HDL-C), while were separately adjusted for glucose and HbA_1c_%. Analysis of MPs, EMPs with their ratios, and protein content within MPs was done by Friedman test followed by Dunn's Multiple Comparison Test. For analysis of the angiogenesis profiler assay data, SPSS version 25.0 was used. Mean, and standard deviation (SD) and the median and interquartile range (IQR) were reported to summarize the microparticles data by patient type. The non-parametric Kruskal–Wallis’s test was used on data since the assumptions of normality were violated when tested using a Kolmogorov–Smirnov Test. Statistical analysis was carried out using GraphPad Prism V5 and STATA version 25.0. p < 0.05 was considered statistically significant.

## Results

### Characteristics of study subjects

Eighty research participants (43 women and 37 men) were consecutively recruited in this study. The main characteristics and biomedical parameters are presented in Table 1. Research participants had a mean age of 56.05 years, the oldest being patients with CCAD. Diabetes duration was considered the highest among participants having T2D and CCAD with an average of 19.75 ± 10.07 years (Mean ± SD). This group also has a higher systolic blood pressure level than the rest of the groups. However, patients with an ACS suffer from poor diabetes control with a significantly higher level of HbA_1c_ (average 8.57%) and glycemia (11.43 mmol/L) compared to those with CCAD and the rest of the participants. No significant differences in BMI and lipid profile were noticed among the four groups, but patients with ACS smoked the most. Almost all patients with T2D and CAD (CCAD or ACS) suffered from hypertension and dyslipidemia and were on ACEi/ARB medications, in addition to aspirin. Interestingly, no documented peripheral arterial disease was found in any of the different groups.

**Table 1:** Characteristics, biomedical parameters, risk factors, and treatment of all research participants

Characteristics	Controls (n = 20)	T2D without cardiovascular disease (n = 20)	T2D with CCAD (n = 20)	T2D with ACS (n = 20)
Characteristics and biomedical parameters of study subjects				
Gender (M/F)	4/16	6/14	12/8	15/5
Age, years (SD)	45.6 (11.6)	58.7 (8.724)	60.6 (9.344)	59.3 (10.96)*
Diabetes duration, years (SD)	N/A	18.17 (11.05)	19.75 (10.07)	14.94 (10.27)*
BMI, Kg/m^2^ (SD)	33.04 (9.526)	32.84 (6.664)	32.24 (10.13)	30.3 (5.875)
Systolic BP, mmHg (SD)	121.5 (15.55)	129.5 (18.79)	132.5 (17.31)	127.6 (20.3)*
Diastolic BP, mmHg (SD)	73.35 (9.167)	73.9 (9.008)	74 (9.674)	72.3 (9.652)
HbA_1c,_ % (SD)	5.611 (0.4841)	8.4 (2.457)	7.98 (1.684)	8.57 (2.067)*
Glucose, mmol/L (SD)	5.31 (0.9296)	10.21 (6.374)	10.93 (6.986)	11.43 (5.157)*
Serum Creatinine, µmol ( SD)	63.55 (10.49)	68.65 (19.18)	93.06 (61.34)	76.78 (18.38)
Total Cholesterol, mmol/L (SD)	5.278 (0.9808)	4.312 (0.9357)	4.087 (1.087)	4.166 (1.411)
Triglycerides, mmol/L (SD)	1.372 (0.6748)	1.516 (0.5058)	2.356 (2.564)	1.714 (1.071)
HDL-C, mmol/L (SD)	1.456 (0.4329)	1.184 (0.2444)	1.148 (0.2322)	1.084 (0.3862)
LDL-C, mmol/L (SD)	3.271 (0.8789)	2.452 (0.8472)	1.994 (0.8062)	2.304 (1.099)
Risk factors and treatments				
Current Smoker (%)	5/20 (25%)	3/20 (15%)	4/20 (20%)	8/19 (42.1%)*
Hypertension (%)	3/20 (15%)	15/20 (75%)	18/20 (90%)	17/20 (85%)
Dyslipidemia (%)	5/20 (25%)	17/20 (85%)	20/20 (100%)	19/20 (95%)
Peripheral Arterial Disease (%)	0/20 (0%)	0/20 (0%)	0/20 (0%)	0/20 (0%)
Coronary Artery Disease (%)	0/20 (0%)	0/20 (0%)	20/20 (100%)	20/20 (100%)
Stroke (%)	0/20 (0%)	0/20 (0%)	0/20 (0%)	1/20 (5%)
Statins (%)	5/20 (25%)	17/20 (85%)	20/20 (100%)	18/19 (94.74%)*
ACEi/ARB (%)	1/20 (5%)	11/20 (55%)	17/20 (85%)	18/19 (94.74%)*
Insulin (%)	NA	10/20 (50%)	11/20 (55%)	9/19 (47.37%)
Oral anti-diabetic agents (%)	NA	19/20 (95%)	14/20 (70%)	17/19 (89.47%)
Aspirin (%)	0/20 (0%)	2/20 (10%)	20/20 (100%)	19/19 (100%)*

Data is presented using mean (SD) or number (percentage)

*T2D* Type 2 diabetes, *ACS* Acute Coronary Syndrome, *CCAD* Chronic coronary artery disease, *BMI* Body Mass Index, *N/A* not applicable, *BP* Blood Pressure, *SD* Standard Deviation

*p < 0.05 for comparison among the three groups of patients with type 2 diabetes

### Evaluation of EMPs

**Fig. 1:**
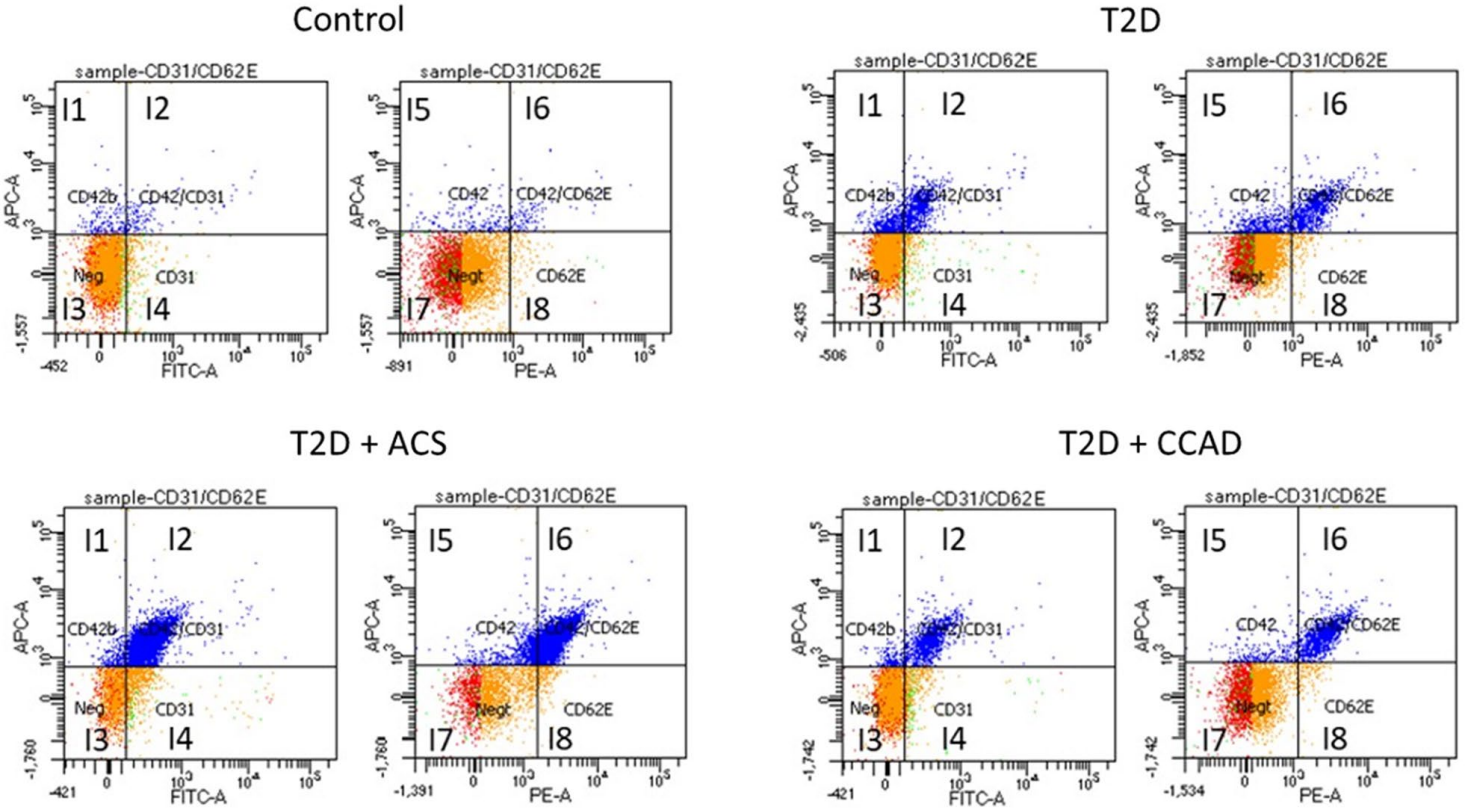
Representative EMPs profiles in controls and T2D with and without CAD, as analyzed by flow cytometry. I1 quadrant shows the number of CD42b positive MPs, I2 shows CD42 positive/CD31 negative MPs, I3 shows CD42b/CD31 negative MPs, and l4 shows CD42 CD31 positive MPs. I5 shows CD42 the number of CD42b positive MPs, I6 shows CD42 positive/C62E negative MPs, I7 shows CD42b/CD62E negative MPs, and l8 shows CD62E positive MPs

Isolated EMPs were analyzed for their expression of CD42 (platelet marker), CD62E (E selectin expressed in endothelial cells), and CD31 (endothelial cell marker). We determined the levels of circulating EMPs based on CD42 and CD31 staining. We further investigated the expression of CD62E in these MPs to assess the apoptosis/ activation states of the evaluated EMPs. Figure 1 shows representative scatter plots of circulating CD42-CD31 + EMPS and CD42–CD62 + EMPs in non-diabetic controls compared to patients with diabetes with and without CAD.

**Fig. 2:**
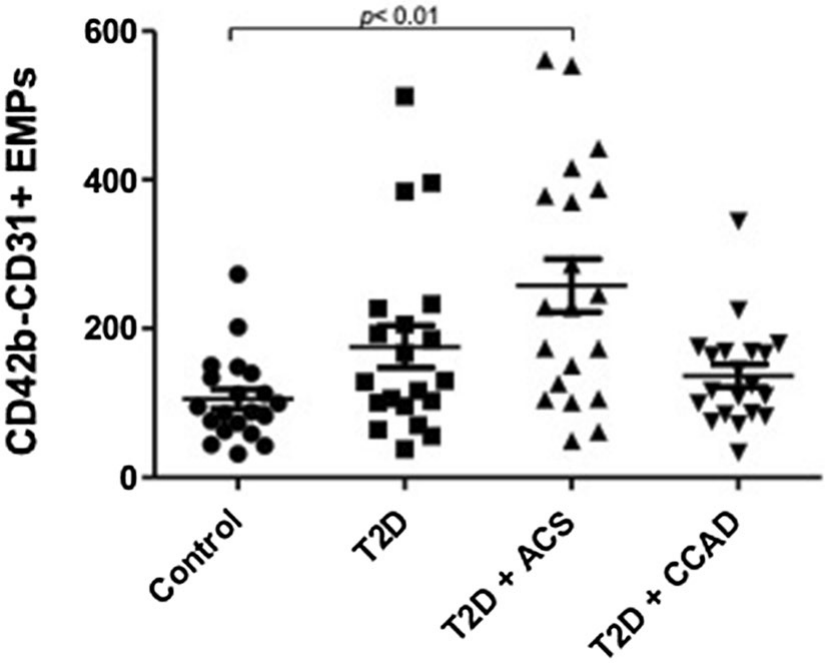
Levels of CD42–CD31 + EMPs in plasma of healthy subjects, diabetes patients (without macrovascular disease), diabetes patients with ACS, and diabetes patients with chronic CAD subjects (n = 20). P values are shown in the figure

Levels of CD42–CD31 + EMPs were significantly increased over two-fold in diabetes patients with ACS when compared to healthy subjects (257.5 ± 35.58 vs. 105.7 ± 12.96; respectively, p < 0.01). On the other hand, there was no significant difference when comparing healthy subjects with diabetes or diabetes patients with CCAD (Fig. 2). Similarly, no difference was seen between T2D patients with ACS versus CCAD.

**Fig. 3:**
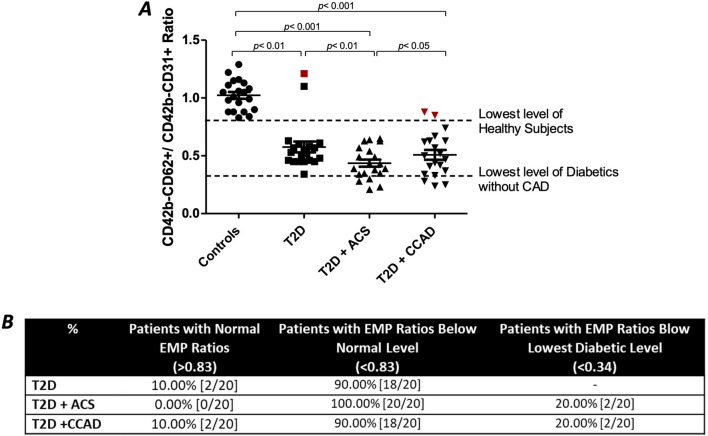
**A** The ratio of CD42–CD62 + /CD42–CD31 + EMPs in plasma of healthy non-diabetes subjects, diabetes subjects (without CVD), diabetes with ACS, and diabetes with chronic CAD subjects (n = 20). **B** Table showing patients categories in accordance with EMPs ratios, categorized into three groups: (1) within normal range, (2) below the normal range, and (3) below the lowest diabetic ratio

Furthermore, a ratio of CD42-CD62 + / CD42-CD31 + was calculated as an indication of EMPs activation/apoptosis state (Fig. 3A). A high ratio indicates activated EMPs, while a low ratio is associated with apoptotic EMPs. The mean (± SEM) of EMPs ratio in the control group was 1.023 ± 0.029 with the lowest level of 0.83. All studied disease groups showed ratios lower than the minimum ratio seen in the control group (p < 0.01). Among patients with diabetes, those with CCAD (0.51 ± 0.04, n = 20) and those with ACS (0.44 ± 0.03, n = 20) had lower values than healthy T2D patients without CVD (1.02 ± 0.03, n = 20). The mean ratios in patients with ACS (0.44 ± 0.03) were significantly lower than that of the diabetes group without complications (0.58 ± 0.05, p < 0.05). In addition, there was a significant reduction in the mean ratios between ACS and CCAD subjects (0.44 ± 0.03 and 0.51 ± 0.04 respectively, p < 0.05). On the other hand, there was a non-significant difference between healthy diabetes patients and those with CAD (0.58 ± 0.05 and 0.51 ± 0.04, respectively). Figure 3B categorizes patients’ groups according to their EMPs ratios into three categories: (1) patients with normal EMPs ratios (> 0.83), (2) patients with EMPs ratios below the normal level (< 0.83), and (3) patients with EMPs ratios below the lowest diabetic ratio (< 0.34). The table shows that 10% of healthy diabetes and diabetes with CCAD groups had normal EMPs ratios, and 20% of diabetes with ACS and CCAD had below the lowest diabetic EMPs ratio.

We further investigated the association between EMPs ratio and the basic characteristics of the entire population of the study, including BMI, blood pressure, glucose, HbA_1c_, triglycerides, LDL-C, and HDL-C (Table 2). EMPs ratio showed a moderately strong negative correlation with levels of glucose (r = -0.433, p < 0.0001) and HbA_1c_ (r = -0.459, p < 0.0001). Additionally, EMPs ratios showed a moderately strong positive correlation with total cholesterol (r = 0.4926, p < 0.0001) and LDL-C (r = 0.493, p < 0.0001), and a weak positive correlation with HDL-C (r = 0.305, p = 0.016). There was no significant association between EMPs ratios and BMI, systolic or diastolic pressure, or triglycerides.

**Table 2:** Correlation between EMPs Ratio and essential characteristics of the studied groups

Correlation between EMPs Ratio and baseline characteristics of the studied groups		
Variable	Spearman	P value
BMI	0.141	0.215
Diastolic blood pressure	0.025	0.826
Systolic blood pressure	− 0.021	0.851
Glucose	− 0.433	< 0.0001
HbA_1C_ %	− 0.459	< 0.0001
Total Cholesterol	0.493	< 0.0001
Triglycerides	0.027	0.831
LDL-C	0.493	< 0.0001
HDL-C	0.305	0.016

Correlations were performed using the non-parametric Spearman rank correlation test

P < 0.05 was considered significant

We have further performed a multiple linear regression analysis to assess the relationship between EMPs ratio and the baseline characteristics of the studied groups while adjusting for glucose-related variables (namely: blood glucose and HbA_1C_ %) (Table 3). The normality of residuals was assessed using QQ plots in addition to Anderson Darling, D'Agostino-Pearson omnibus, Shapiro Wilk, and Kolmogorov Smirnov tests. These tests indicated that the residuals were normally distributed for all variables’ combinations. The multiple linear regression analysis revealed that total cholesterol (R^2^ = 0.4, β = 0.12, |t|= 4.73 and *p* < 0.0001), LDL-C (R^2^ = 0. 38, β = 0.13, |t|= 4.30 and *p* < 0.0001), and HDL-C (R^2^ = 0.26, β = 0.24, |t|= 2.56 and *p* = 0.013) showed positive and significant association with EMPs ratio. These results were in line with the correlation results shown in Table 2.

**Table 3:** Multiple linear regression analysis of EMPs Ratio (dependent variable) and baseline characteristics of the studied groups, adjusted to glucose and HbA_1C_

Multiple linear regression analysis of EMPs Ratios and baseline characteristics of the studied groups, adjusted to glucose and HbA_1C_ %				
Variable	R^2^	β Coefficient	|t|	*p* value
BMI	0.21	0.0036	1.013	0.314
Diastolic BP	0.20	0.0008	0.240	0.811
Systolic BP	0.20	0.0001	0.082	0.935
Total Cholesterol	0.41	0.1170	4.728	< 0.0001
Triglycerides	0.18	0.0043	0.152	0.880
LDL-C	0.38	0.1323	4.295	< 0.0001
HDL-C	0.26	0.2419	2.562	0.013

### Characterization of MPs’ protein content and angiogenic profile

The protein content in the total circulating population of MPs was quantified using Bradford assay in all four groups (Additional file 1: Fig. S2A). There was no significant difference in the protein content of MPs between the studied groups. However, the distribution of MPs proteins was different between each group (Additional file 1: Figure S2B). This indicates that protein profile/ expression is variable in relation to the disease state. Due to these variations, we further investigated the variation in the content through a protein profiler array. Furthermore, the origin of the studied particles was evaluated by western blot of cell-specific markers (Additional file 1: Figure S2C). In addition to the previously identified endothelial markers, western blot analysis of the complete portion of the isolated MPs showed a positive expression of the monocyte markers CD14 and CD16 and the platelet marker CD41.

**Fig. 4:**
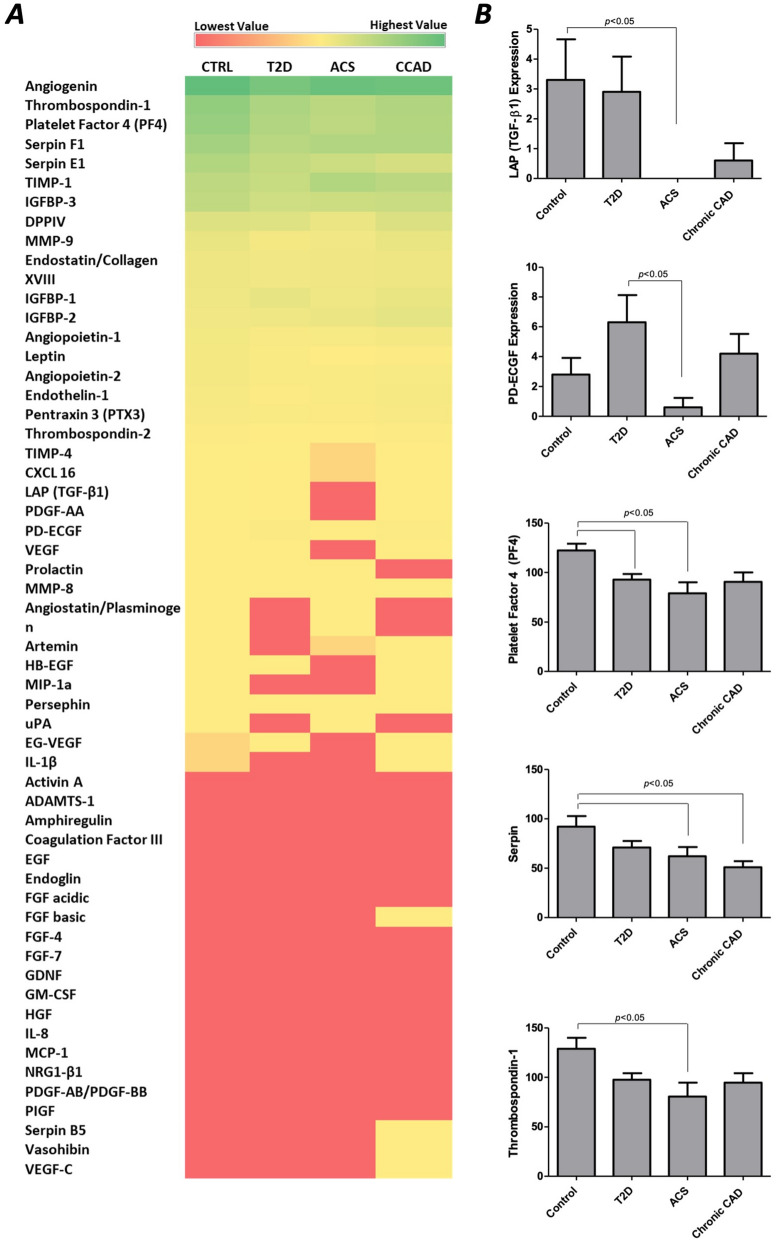
**A** Heat map shows mean values of expressed angiogenic factors, where red represents low expressed factors, yellow shows moderate expression, and green represents high expression. **B** five of the tested angiogenic factors showed a significant difference between the study groups and were plotted separately. The plotted figures were further analyzed by ANOVA followed by Bonferroni posttest, and p < 0.05 was considered significant. P values are shown on the figure

Data extracted from the angiogenesis profiler assay were analyzed and represented as a heat map, showing the average expression of each protein in each patient group (Fig. 4A), and the detailed expression of the studied angiogenic factors is shown in supplementary materials (Additional file 1: Table S1 and Fig. S3). Out of the 55 analyzed angiogenesis-related proteins, five factors were differentially expressed across the study groups. These include LAP (TGF-β1), PD-ECGF (also known as Thymidine Phosphorylase TYMP), platelet factor 4 (PF4), serpin E1, and thrombospondin 1 (THBS1). LAP (TGF-β1) showed a dramatically low expression in MPs isolated from patients with T2D and ACS. PD-ECGF was significantly lower in MPs isolated from ACS when compared to patients with T2D without CAD (0.600 ± 2.8 and 6.300 ± 8.2 respectively, *p* < 0.05). PF4 was significantly reduced in patients with diabetes without *versus* those with ACS (92.800 ± 26.1, 79.000 ± 50.3 respectively, *p* < 0.05), but not with CCAD (90.700 ± 41.8). Serpin E1 was significantly reduced in patients with diabetes with ACS and CCAD (62.200 ± 41.4 and 50.900 ± 28.3, respectively) when compared with controls (92.200 ± 47.3, *p* < 0.05) but was not affected in patients without CAD (71.000 ± 29.5). THBS1 was significantly reduced in T2D with ACS (80.700 ± 62.8) when compared to controls (129.100 ± 49.6, *p* < 0.05) (Fig. 4B). Factors including FGF basic, Serpin B5, Vasohibin, and VEGF-C were only identified in CCAD MPs while undetermined in MPs from the other three groups. These findings might indicate a specific and unique MPs composition in relation to the disease type and stage.

**Fig. 5:**
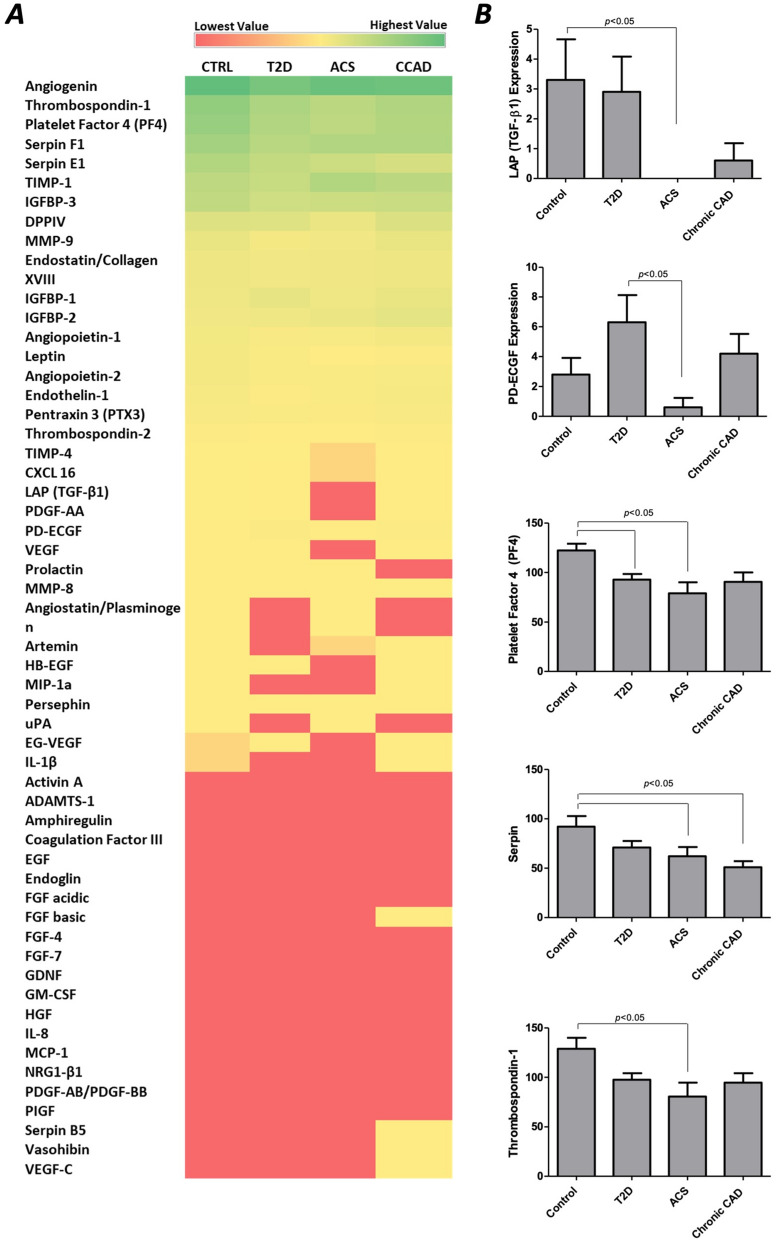
**A** Heat map shows mean values of expressed angiogenic factors, where red represents low expressed factors, yellow represent moderate expression, and the green represents high expression. **B–D** factors showing significant differences between the study groups were plotted separately. **B** shows differentially expressed proteins between control and all diabetic groups (compiled), **C** shows comparisons between T2D alone against all CAD groups (assembled), and **D** shows ACS against CCAD. P values are shown on the figure

To further understand the effect of the disease state on the expression of the studied angiogenic factors in the isolated MPs, data were regrouped to compare: (i) healthy controls against all groups of T2D (including T2D, T2D + ACS, and T2D + CCAD), (ii) healthy diabetics without CVD (T2D) against T2D patients with coronary artery disease (T2D + ACS and T2D + CCAD), and (iii) diabetics with acute coronary syndrome (T2D + ACS) against diabetes with chronic CAD (T2D + CCAD) (Fig. 5). These comparisons revealed that in all T2D groups, 6 factors were differentially expressed when compared to healthy controls, including Angiopoietin-2 (*p* = 0.018), Leptin (*p* = 0.038), PF4 (*p* = 0.001), Serpin E1 (*p* = 0.001), Serpin F1 (*p* = 0.027) and THBS1 (*p* = 0.001) (Fig. 5B). These factors were all significantly reduced in MPs isolated from the T2D groups. Comparisons of healthy diabetics and diabetics with CAD (both ACS and CCAD) revealed a significant reduction in EG-VEGF (*p* = 0.022*)*, LAP (TGF-b1) (*p* = 0.007*)*, Leptin (*p* = 0.04), PD-ECGF (*p* = 0.038), Serpin F1 (*p* = 0.038) and TIMP-4 (*p* = 0.02), in all CAD patients while TIMP-1 was significantly increased in this group (*p* = 0.023) (Fig. 5C). Finally, a comparison between ACS and CCAD showed a significant decrease in DPP-IV expression in ACS patients (*p* = 0.033) (Fig. 5D).

**Fig. 6:**
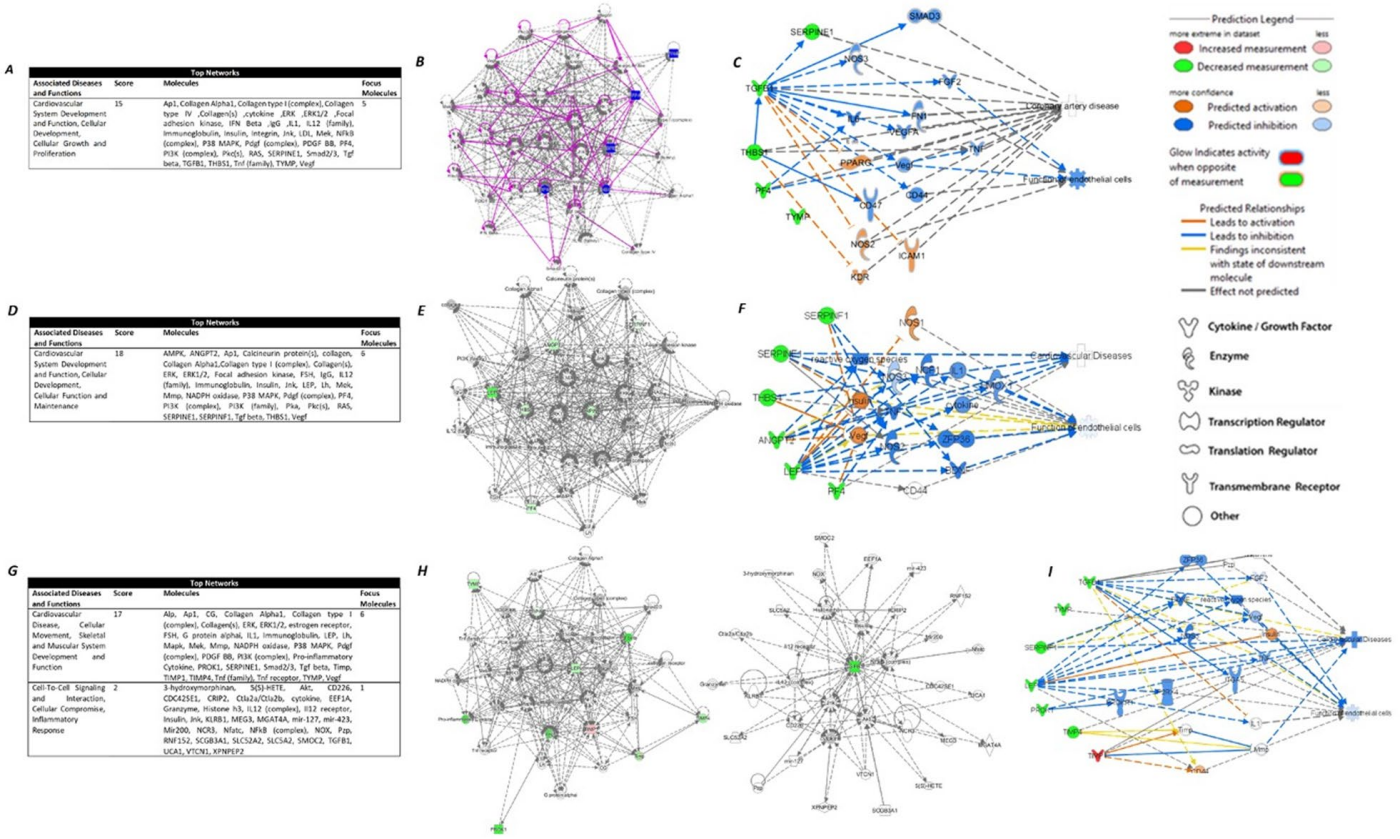
Ingenuity Pathway Analysis (IPA) of the differentially expressed angiogenic factors. The top panel shows data analysis of all compared study groups. The middle panel shows comparisons of healthy controls against all T2D patients compiled (with and without CAD), and the bottom panel shows comparisons between healthy T2D and the ones with CAD compiled. **A**, **D**, **G** Top networks and associated diseases/functions, with scores and associated molecules, obtained from IPA. **B**, **E**, **H** Representation of the most highly rated network in IPA. Highlighted are the differentially expressed molecules and relationships where the interaction is direct. **C**, **F**, **I** IPA prediction of the interactive network associated with the differentially expressed proteins in relation to CAD and their effect on endothelial function. Green: decreased measurement, blue: predicted inhibition, Orange: predicted activation. Straight-line: direct interaction, segmented line: indirect relationship. Data was generated by QIAGEN’s Ingenuity ® Pathway Analysis application (www.qiagen.com/ingenuity)

IPA analysis was performed to investigate further the networks and functions affected by the differentially expressed proteins from the profiler array comparisons between the four groups (Fig. 6A–C). The top molecular network identified by IPA was associated with the following diseases and functions: cardiovascular system development and function, cellular development, cellular growth, and proliferation, with five focus molecules and a significance score of 15 (Fig. 6A). The top affected network by the patterns of the differentially expressed proteins is shown in Fig. 6B. A prediction network was created to understand further the effects of these expression patterns on endothelial function (Fig. 6C). A reduction in the expression of the five differentially expressed molecules (mainly TGF-β1) is predicted to cause an inhibition of critical pathways, which leads to endothelial dysfunction. These include NOS3, FGF2, and VEGFA. Other molecules are expected to be activated, including KDR, ICAM1, NOS2, and PPARG, which also play a role in the pathogenesis of CAD.

The regrouped data was also analyzed using IPA (Fig. 6D–I). The differentially expressed factors identified in all T2D groups compared to healthy controls were also associated with Cardiovascular System Development and Function, Cellular Development, Cellular Function, and Maintenance, with six focus molecules (Angiopoietin-2, Leptin, PF4, Serpin E1, Serpin F1, and THBS1) and a significance score of 18 (Fig. 6D). The top affected network by the patterns of these proteins is shown in Fig. 6E. The identified patterns of the expression of the proteins are predicted to inhibit endothelial cell function and affect key signaling pathways, including NOS3, reactive oxygen species, insulin, and VEGF (Fig. 6F). However, some of the effects showed discrepancies with the IPA database (yellow lines).

Similarly, the differentially expressed patterns between patients with T2D and CAD – both chronic and acute- in comparison with healthy diabetics showed an effect on Cardiovascular System Development and Function, Cellular Development, Cellular Function, and Maintenance with a score of 17 and 6 focus molecules (EG-VEGF, LAP (TGF-b1), Leptin, PD-ECGF, Serpin F1, and TIMP-4), in addition to functions associated with Cell-To-Cell Signaling and Interaction, Cellular Compromise, Inflammatory Response, with a score of 2 and 1 focus molecule (DPP-IV) (Fig. 6G). The top affected networks by the patterns of these proteins are shown in Fig. 6H. Prediction networks have also demonstrated an expected impact on endothelial cell function but to a lesser extent to the previously described (Fig. 6I, inhibition of endothelial cell function indicated in light blue). Some affected pathways include NOS3, reactive oxygen species, insulin, VEGF, TNF and TIMP.

## Discussion

Circulating EMPs could have a predictive role in diabetic CVD, as they can reflect the imbalance between angiogenesis and apoptosis and its possible involvement in the pathogenesis of CVD [23, 24]. In the present study, we quantified the release of circulating EMPs by subjects affected by diabetes in combination with or absence of CAD. To that end, we enumerated CD42-CD31 + MPs and CD42-CD62E + MPs and calculated the ratio between these groups to define the activation/apoptosis states of endothelial-releasing MPs. We demonstrated that EMPs are increased in T2D patients with ACS. We further confirmed that the ratio of CD42-CD62 + / CD42-CD31 + EMPs was reduced in all diabetic groups, reflecting a release by apoptotic endothelial cells. We further studied the content of MPs in terms of the release of angiogenic factors. We found a differential expression of 5 factors, including LAP (TGF-β1), PD-ECGF, platelet factor 4 (PF4), serpin E1, and THBS1.

MPs are released by variable cell types and are distinguished by the expression of specific cell surface antigens. EMPs, for example, are distinguished from other MPs by their expression of the platelet endothelial cell adhesion molecule CD31 in the absence of the platelet marker CD42b. These MPs can be further categorized into apoptosis-induced and activation-induced EMPs, relying on their expression of the inducible endothelial marker CD62E [16, 19, 20]. Several studies showed an increased release of CD42b − CD31 + EMPs in diseases involving endothelial dysfunction such as diabetes, CVD [25], and arterial hypertension [26]. The current study showed that CD42b − CD31 + EMPs were elevated in T2D with ACS, but not chronic CAD. This is in line with previous findings [27, 28].

One of the main hallmarks of diabetes is endothelial injury and dysfunction [29], resulting in vascular dysfunction and contributing to diabetes vascular complications [30, 31]. It has been previously suggested that exposure to hyperglycemia results in a switch of endothelial cells to an apoptotic phenotype [32–37], which accelerates the pathogenesis of diabetes and contributes to atherosclerosis. In this context, we showed that the ratio of CD42–CD62 + /CD42–CD31 + EMPs was reduced in all diabetic groups, reflecting a release by apoptotic endothelial cells. Interestingly, we found a significant reduction in the ratio of CD42–CD62 + /CD42–CD31 + EMPs between healthy T2D and diabetics suffering from ACS, but not chronic CAD. This could be attributed to innate repair mechanisms in chronic CAD patients or their response to medication. This observation is consistent with the notion that endothelial dysfunction might be reversible at every stage of atherosclerosis [38]. It has been previously shown that coronary endothelial dysfunction is frequently manifested in patients with ACS and is reversible in most cases within six months [39]. This might explain the reduction in the release of apoptotic EMPs due to the recovery of endothelial cells in chronic CAD.

We further sought to investigate the association between EMPs ratios and the factors that determine diabetes severity and progression to CVD, such as glucose, HbA_1c_, lipid profile in addition to BMI and blood pressure. We observed an inverse relationship between EMPs ratios and levels of glucose and HbA_1C._ These observations align with previous findings indicating a strong relationship between poor glycemic control and severe endothelial dysfunction, which is also correlated with the progression of atherosclerosis [40]. However, other confounding factors not included in our model might have contributed to the observed effects. We also found a positive association between EMPs ratios and total cholesterol and LDL-C levels. Similar observations were obtained when linear regressions adjusted to glycemia and HBA_1C_ were performed. These results are unexpected because oxidized LDL can induce endothelial cell apoptosis through several mechanisms [41]. These findings could be affected by confounding factors or the use of lipid-lowering drugs. A previous study by Nomura et al. has shown that oxidized LDL induces the generation of EMPs and suggests that this increase could predict vascular complications in diabetes [42, 43].

MPs released by activated or apoptotic cells into the circulation carry an array of biomolecules and contribute to the regulation of cellular processes and cell–cell communication. This study showed that the protein content of circulating MPs and their expression pattern was different between the studied groups. This is expected because diabetes alters the function of several cell types such as endothelial cells, platelets, and immune cells [44]. Furthermore, it has been previously suggested that the composition of MPs is influenced by the microenvironment, affecting their phenotypes and functions [45, 46]. Because angiogenesis is essential in developing diabetic vascular complications, we sought to profile the angiogenic factors contained within the isolated circulating MPs from the studied groups. Angiogenesis involvement in CVD development has two sides, a pathological and a therapeutic side [47]. Many studies suggest that angiogenesis contributes to the growth of plaques and their rupture, leading to myocardial infarction [48]. On the other hand, angiogenesis can induce revascularization, re-establish blood flow to ischemic tissues, and improve myocardial functions [49, 50]. Previous reports of the angiogenic potential of MPs have been controversial as well. It has been suggested that MPs could carry both pro and antiangiogenic factors and that their angiogenic effects are determined by the MPs origin [51–59]. In the current study, we have identified angiogenic regulators that were differentially expressed in the circulating MPs of the studied groups. Initial analysis of all four groups showed a differential expression of LAP (TGF-β1), THBS1, PF4, serpin E1, and PD-ECGF, and others that were only expressed in chronic CAD MPs, including FGF basic, Serpin B5, Vasohibin, and VEGF-C.

TGF-β1 is secreted by immune cells and has an essential role in regulating vascular development and angiogenesis [60]. TGF-β1 has an inhibitory effect on endothelial cells and has been suggested to modulate angiogenesis directly and indirectly through the regulation of immune cells [60, 61]. TGFβ-1 is expressed as an inactive pro-protein composed of the latency-associated peptide (LAP) and mature TGFβ-1 [62]. Multiple factors, including THBS1, control the activation of TGFβ-1. THBS1 is found in platelet α-granules and is released by activated platelets [62]. It has been suggested that THBS1 plays a role in the pathogenesis of atherosclerosis, where its absence was shown to accelerate atherosclerosis in apolipoprotein E deficient mice [63]. Additionally, THBS1 was shown to reduce vascular remodeling following myocardial infarction [64]. In the current work, we have demonstrated that the expression of LAP (TGF-β1) and THBS1 was significantly reduced in MPs of ACS patients compared to controls. These results align with previous findings showing a reduction of TGF-β1 serum levels in atherosclerosis, CAD, and ischemic heart disease. [65–68]. THBS1 has been shown to increase 24 h acutely following myocardial infarction [69]. The increase in THBS1 in the heart ischemia model was linked to scaring and perivascular fibrosis [64, 70]. A reduction in THBS1 following percutaneous coronary intervention has been previously associated with major adverse cardiac events.

PF4 and Serpin E1 also inhibit angiogenesis, while PD-ECGF is an angiogenesis activator. PF4 is a chemokine known to inhibit the interaction of angiogenesis growth factors with cell receptors and inhibit endothelial cell proliferation and migration [71]. Serpin E1 is a serine protease inhibitor mainly produced by endothelial cells and is the key inhibitor of plasminogen activators [72]. Plasminogen activators convert plasminogen into plasmin, which has a vital role in fibrinolysis, activation of MMP-s (a pre-request to angiogenesis), and conversion of TGF-β from a latent to an active form. Serpin E1 was previously identified in MPs from autophagic/apoptotic serum-starved human endothelial cells [73]. PD-ECGF is an angiogenesis activator that promotes endothelial cells growth and chemotaxis [74]. In terms of their plasma levels in relation to CAD, both PF4 and serpin E1 were previously found to be induced [75–77]. PF4 is induced by atherosclerotic lesions and myocardial ischemia, while serpin E1 release is associated with impaired basal fibrinolytic activity [75, 77]. PD-ECGF was previously found to be expressed in the atherosclerotic plaque, and its expression was related to the number of lesional microvessels [78]. The current study reports that PF4 was significantly reduced in MPs of patients with diabetes without CAD and those with ACS, but not CCAD. We also observed a significant reduction of Serpin E1 in MPs from diabetics with ACS and established CAD. Additionally, we observed a decrease of PD-ECGF in MPs from patients with diabetes with ACS and a trend towards an increase in T2D patients without and with chronic CAD.

Further analysis of the regrouped angiogenesis profiler array data showed that in addition to a reduction in PF4, Serpin E1, and THBS1, other factors were also reduced in MPs from all diabetic subjects compiled (with and without CAD), including Angiopoietin-2, Leptin, and Serpin F1. Angiopoietins act together with VEGF to regulate angiogenesis, where Angiopoietin-1 promotes endothelial cell survival and protects from VEGF’s effects on permeability. In contrast, Angiopoietin-2 acts as an antagonist to these effects in the absence of VEGF, thus limiting vasculogenesis [79, 80]. In the presence of VEGF, however, Angiopoietin-2 stimulates angiogenesis [80]. Angiopoietin-2 was previously shown to increase in diabetics, and this increase was controllable with Intensive multifactorial intervention [79]. Leptin is another angiogenic stimulator that acts synergically with VEGF and FGF-2 to promote angiogenesis [81]. Serpin F1, on the other hand, is a strong antiangiogenic factor that also possesses other cardioprotective antithrombotic and anti-inflammatory characteristics [82, 83]. Serpin F1 was also identified as a potent inhibitor of endothelial progenitor cell-mediated vasculogenesis [84].

Comparing healthy diabetics to those with cardiovascular complications revealed a reduction in EG-VEGF and TIMP-4 (in addition to LAP (TGF-b1), Leptin, PD-ECGF, and Serpin F1) in all patients with diabetes with CAD while TIMP-1 was significantly increased. EG-VEGF (also known as prokineticin 1) is a potent angiogenic factor selective for endocrine endothelium with functional similarities to VEGF [85].In addition to promoting the proliferation of adrenal cortex endothelial cells, its receptor (PKR1) was found to have some cardioprotective effects. PKR1 was found to mediate cardiac angiogenesis and protect from oxidative-stress-associated apoptosis, thus preventing heart failure [86]. Tissue inhibitors of metalloproteinases (TIMPs) are inhibitors of angiogenesis [87]. TIMP-4 is highly expressed in the cardiac tissue [88] and was reduced in myocardial infarction and ischemic cardiomyopathy [89–91]. Dysregulation of TIMP-4 is associated with CVD [91, 92]. TIMP-1 is suggested to block the release of matrix-bound angiogenic factors or to inhibit endothelial response to pro-angiogenic factors [93]. TIMPs are also critical effectors in cardiac remodeling through their interaction with matrix metalloproteinases [94].

DPP-IV inhibition exerts a protective effect on endothelial function, increases flow-mediated dilation in vessels, reduces inflammation and oxidative stress95. In our study, the expression of DPP-IV was lower in ACS patients when compared to patients with chronic CAD, which could be the down-regulation consequence of acute myocardial and vascular injury observed in ACS. Previous studies have shown an increase of DPP-IV serum levels in CAD with and without T2D and found a possible correlation between DPP-IV levels and cardiac dysfunction [96, 97] DDP-IV is highly expressed by endothelial cells and was shown to increase in cells treated with high glucose [98, 99]. DPP-IV inhibitors are oral drugs used to treat T2D, which are known to reduce – albeit moderately- HbA_1c_ without increasing weight gain or hypoglycemic events [100]. Despite the presence of in-vitro studies showing a protective effect of DPP-IV inhibitors in cardiovascular physiology, cardiovascular outcomes of DPP-IV inhibitors failed to show any protection from this family of drugs [101], and some data suggest that heart failure events might be increased with DPP-IV inhibitors [102].

Altogether, our findings indicate an imbalance in the expression of angiogenic factors in diseased MPs, with a probable shift towards a pro-angiogenic phenotype. It is well known that the angiogenesis process is regulated by a balance between the activators and inhibitors. Under normal conditions, this balance is tilted towards inhibition which restrains abnormal vessel formation [103]. The imbalance of the expression of angiogenic factors in MPs could indicate their involvement in the pathological angiogenesis process in diabetes and CAD.

We acknowledge the presence of some limitations in our study. First, methods used for the isolation of EMPs are not yet standardized; there is a need to establish a standardized method to isolate EMPs with high purity and specificity. Our study relied on the use of CD31 and CD42, which were shown to be abundant and sensitive markers for the isolation of EMPs, as several groups have previously established. In combination with CD62E + , these markers allowed the recognition of the activation and apoptosis status of the identified population, as was also done in several previous studies by us and others. It has been established that increased constitutive antigens reflect apoptosis on EMPs such as CD31. In activation, the expression of these antigens is not affected but is combined with an enhanced expression of inducible antigens such as CD62E [104–107]. Combining these markers with other apoptosis markers could provide a better understanding of the state of the cells and the nature of endothelial injury [104]. Despite that, our strategy lacked the determination of other apoptosis markers such as annexin V. Furthermore, among the markers to characterize EMPs, phosphatidylserine (PhtdSer) is a major procoagulant phospholipid and might play a major role in disease evolution [108]. Its absence is also a limitation of our study and should be performed in subsequent evaluations of the role of EMPs in diabetic cardiovascular complications. Second, we have no certainty of the purity provided by the ExoQuick kits used to isolate microparticles. Therefore, we cannot exclude any contamination by cellular debris. However, we do not think that the level of contamination will affect the conclusion of the study. Third, fluorescence minus one controls were not used for gating, however unstained and isotype negative controls were used in addition to size calibration using polystyrene microspheres [109]. Third, while hyperglycemia has been previously shown to affect the activation/ apoptosis status of EMPs and their quantities, other confounding factors such as comorbidities and medications could have affected the observations reported in this study.

## Conclusion

In conclusion, our data showed that the release of apoptosis-induced EMPs is stimulated in T2D patients in the presence and absence of CAD. This stimulation could be linked to endothelial dysfunction, a hallmark of diabetes and an essential factor in developing CVD. The release of apoptosis-induced EMPs was more prominent in ACS when compared to chronic CAD, indicative of innate repair mechanisms in the chronic phase of the disease. This characteristic could indicate an important feature of the stability and progression of chronic CAD whereby the endothelium function could be progressively restored. Additionally, our results indicate the differential expression of crucial angiogenesis regulators, suggesting an imbalance in the angiogenic activity of diseased MPs compared to regular MPs. Further investigations are required to establish the role of cell-specific MPs in the development and progression of CVD in patients with diabetes.

## Supplementary Information

**Additional file 1:** **Figure S1.** Gating strategy to assess EMPs by flow cytometry. Calibrator standard beads in sizes up to 10 μm were used to quantify size. EMPs were defined as elements in platelet-poor plasma with size <1.5μm in a forward and side scatter plots (A), which expressed the platelet/ endothelium adhesion molecule marker CD31 (PECAM-1) and did not express the platelet-specific glycoprotein Ib marker CD42b (B-C). D. Formula to calculate the absolute counts of the CD31 and CD62 populations. **Figure S2.** A) Protein content of MPs as quantified using Bradford assay. B) Distribution of MPs proteins as estimated by western blot. C) expression of the monocyte markers CD14 and CD16 and the platelet marker CD41. No significant difference was detected between the study groups. L: Ladder, C: Controls, T2D: Type 2 Diabetes, ACS: T2D with Acute Coronary Syndrome, CCAD: T2D with Chronic Coronary Artery Disease. **Figure S3.** Heat maps showing the values of expressed angiogenic factors for each subject in the four different groups. Light blue represents low expressed factors, and dark blue shows high expression. **Table S1.** Angiogenic factors expression. P values are shown in the table. * Significant difference between ACS & T2D, # Significant difference between ACS & Controls. ! Significant difference between T2D & Controls, ƚ Significant difference between CCAD and Controls.

## Data Availability

Data are available upon reasonable request from the lead author.

## Abbreviations

ACS: Acute Coronary Syndrome
CAD: Coronary artery disease
CVD: Cardiovascular disease
EMPs: Endothelial microparticles
T2D: Type 2 diabetes
MPs: Microparticles
