# Supplementary material for: Angiogenic content of microparticles in patients with diabetes and coronary artery disease predicts networks of endothelial dysfunction
Source: Cardiovasc Diabetol. 2022 Feb 2;21:17. doi: 10.1186/s12933-022-01449-0 (PMC8812242; doi:10.1186/s12933-022-01449-0)
Supplement: Supplementary file 1 — Additional file 1: Figure S1. Gating strategy to assess EMPs by flow cytometry. Calibrator standard beads in sizes up to 10 μm were used to quantify size. EMPs were defined as elements in platelet-poor plasma with size <1.5μm in a forward and side scatter plots (A), which expressed the platelet/ endothelium adhesion molecule marker CD31 (PECAM-1) and did not express the platelet-specific glycoprotein Ib marker CD42b (B-C). D. Formula to calculate the absolute counts of the CD31 and CD62 populations. Figure S2. A) Protein content of MPs as quantified using Bradford assay. B) Distribution of MPs proteins as estimated by western blot. C) expression of the monocyte markers CD14 and CD16 and the platelet marker CD41. No significant difference was detected between the study groups. L: Ladder, C: Controls, T2D: Type 2 Diabetes, ACS: T2D with Acute Coronary Syndrome, CCAD: T2D with Chronic Coronary Artery Disease. Figure S3. Heat maps showing the values of expressed angiogenic factors for each subject in the four different groups. Light blue represents low expressed factors, and dark blue shows high expression. Table S1. Angiogenic factors expression. P values are shown in the table. * Significant difference between ACS & T2D, # Significant difference between ACS & Controls. ! Significant difference between T2D & Controls, ƚ Significant difference between CCAD and Controls. [file 12933_2022_1449_MOESM1_ESM.docx]

# Expanded Materials & Methods Angiogenesis profiling

Relative expression of 55 angiogenesis-related proteins was determined using the Human Angiogenesis Proteome Profiler^TM^ Array kit (R&D Systems, Abingdon, UK) following the manufacturer’s instructions**.** After blocking the nitrocellulose membrane, 200μg of protein were loaded to each membrane, followed by incubation overnight at 4ºC. Streptavidin-Horseradish Peroxidase (HRP) was then added to the membrane, followed by the Supersignal^TM^ West Pico chemiluminescence substrate (Thermo-Scientific, Dubai, Emirates) to reveal the bands. Following the optimal exposure time of 30 seconds, pictures/data were collected using ChemiDoc Imaging System and imported to NIH ImageJ software. Pictures of the blots were inverted using ImageJ. The average optical density was determined for the two-duplicated spots representing each of the 55 proteins, followed by background subtraction (background signal was measured from the negative control spots). Median optical density values were determined as the target signal. These values were standardized to the average signal of six positive reference spots of the array.

# List of antibodies

| **Target antigen** | **Vendor or Source** | **Catalog #** |
| --- | --- | --- |
| CD31 | BD Biosciences | 555445 |
| CD62 | BD Biosciences | 551145 |
| CD42b | BD Biosciences | 551061 |
| CD45 | BD Biosciences | 340665 |
| CD14 | BD Biosciences | 555397 |
| CD16 | BD Biosciences | 555406 |
| CD41 | Cell Signaling | 13807s |
| Anti-rabbit IgG, HRP-linked antibody | Cell Signaling | 7074S |
| anti-mouse IgG, HRP-linked antibody | Cell Signaling | 7076S |

# Supplementary Figures:


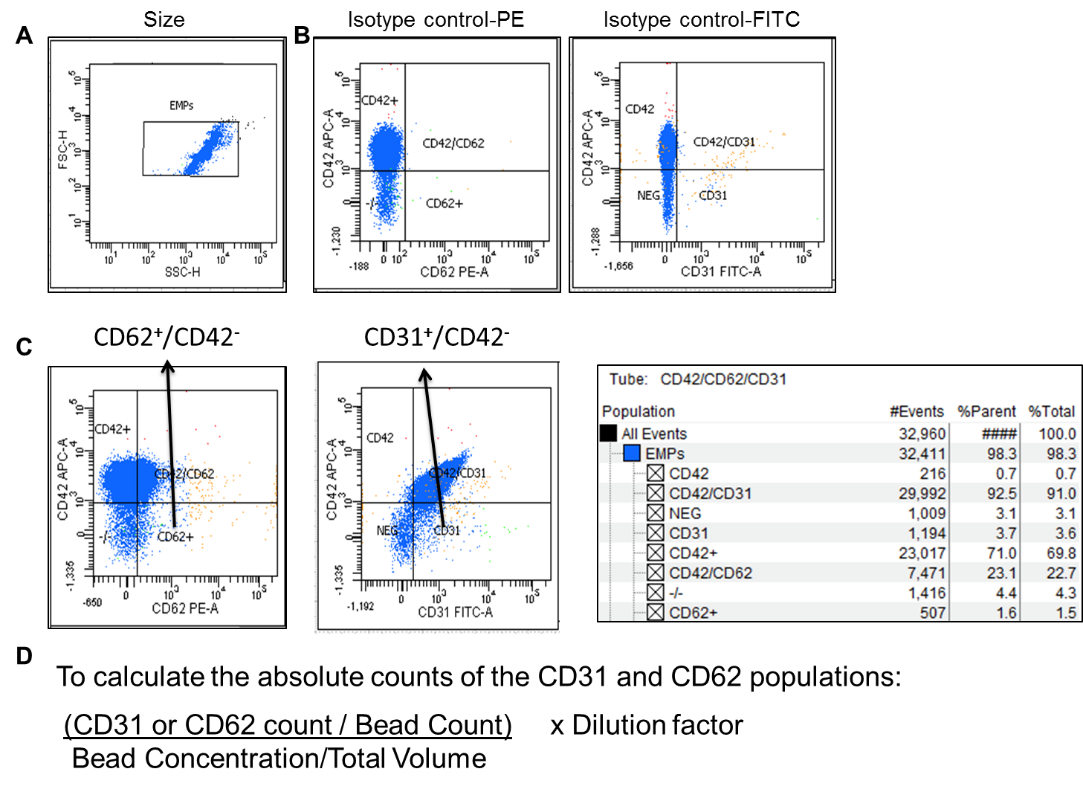


**Figure S1**. Gating strategy to assess EMPs by flow cytometry. Calibrator standard beads in sizes up to 10 μm were used to quantify size. EMPs were defined as elements in platelet-poor plasma with size <1.5μm in a forward and side scatter plots (A), which expressed the platelet/ endothelium adhesion molecule marker CD31 (PECAM-1) and did not express the platelet-specific glycoprotein Ib marker CD42b (B-C). D. Formula to calculate the absolute counts of the CD31 and CD62 populations.


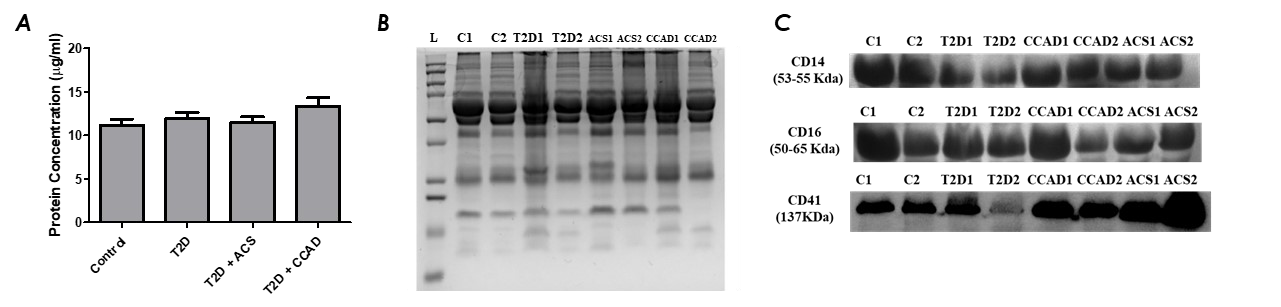


**Figure S2.** A) Protein content of MPs as quantified using Bradford assay. B) Distribution of MPs proteins as estimated by western blot. C) expression of the monocyte markers CD14 and CD16 and the platelet marker CD41. No significant difference was detected between the study groups. L: Ladder, C: Controls, T2D: Type 2 Diabetes, ACS: T2D with Acute Coronary Syndrome, CCAD: T2D with Chronic Coronary Artery Disease.


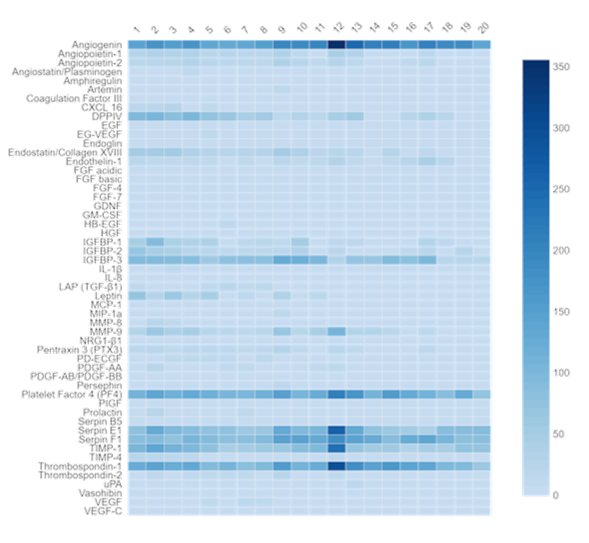

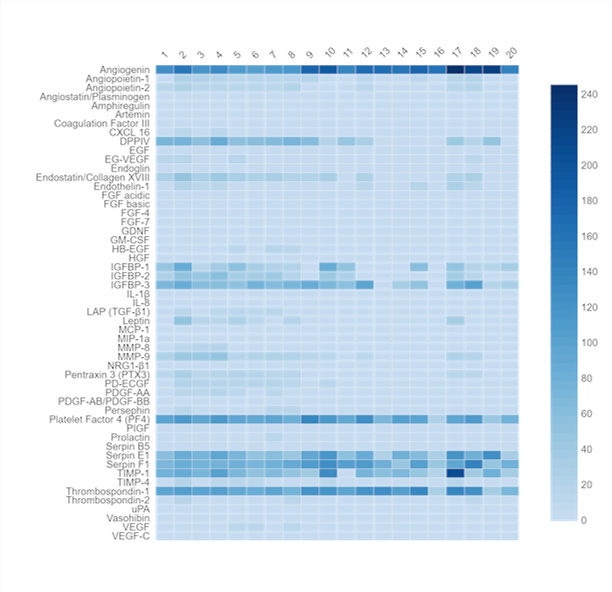

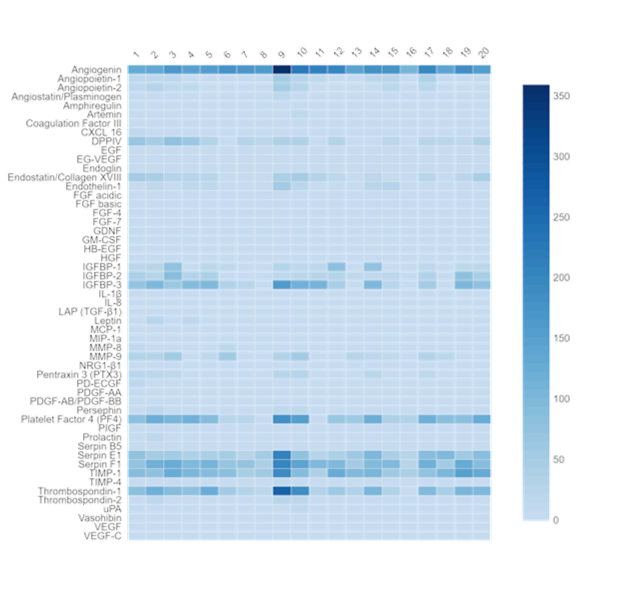

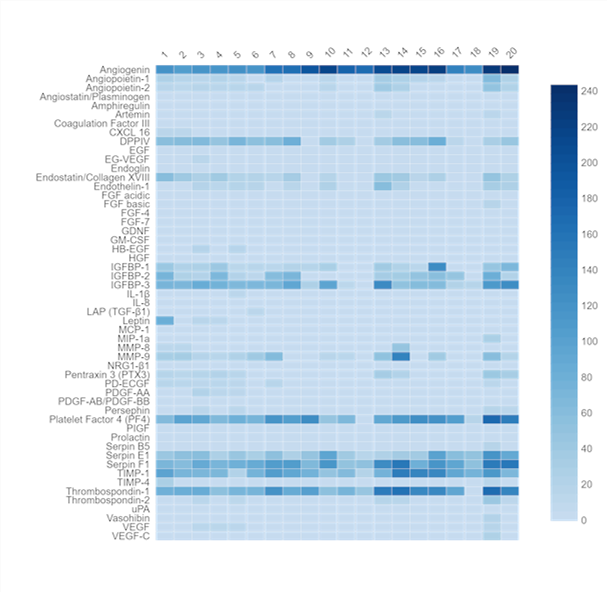


**Control**

**Diabetes**

**Acute**

**Chronic**

**Figure S3.** Heat maps showing the values of expressed angiogenic factors for each subject in the four different groups. Light blue represents low expressed factors, and dark blue shows high expression.

**Table S1:** Angiogenic factors expression. P values are shown in the table. * Significant difference between ACS & T2D, # Significant difference between ACS & Controls. ! Significant difference between T2D & Controls, ƚ Significant difference between CCAD & Controls

| **Angiogenic factors expression in MPS** | | | | | | | | | |
| --- | --- | --- | --- | --- | --- | --- | --- | --- | --- |
|  |  | |  | |  | |  | |  |
|  | **Controls** | | **T2D** | | **T2D + ACS** | | **T2D + CCAD** | | **P-** |
|  |  |  |  |  |  |  |  |  |  |
| **Angiogenic Factor** | **Mean**  **(SD)** | **Median (IQR)** | **Mean**  **(SD)** | **Median (IQR)** | **Mean**  **(SD)** | **Median (IQR)** | **Mean**  **(SD)** | **Median (IQR)** | **value** |
| **Activin A** | 0 (0) | 0 (0-0) | 0 (0) | 0 (0-0) | 0 (0) | 0 (0-0) | 0 (0) | 0 (0-0) |  |
|  |  |  |  |  |  |  |  |  | N/A |
| **ADAMTS-1** | 0 (0) | 0 (0-0) | 0 (0) | 0 (0-0) | 0 (0) | 0 (0-0) | 0 (0) | 0 (0-0) | N/A |
| **Angiogenin** | 184.2  (51.7) | 179.1 (146.7-  202.6) | 157.5  (40.5) | 156.8 (123.1-  177.3) | 175.6  (52.2) | 167.5 (145-  191.4) | 170.1  (46.5) | 166.4 (121.2-  217) | 0.367 |
| **Angiopoietin-1** | 16.3  (14.3) | 16.4 (0-29.6) | 9.5 (8.6) | 11.4 (0-15.9) | 10.1  (14.3) | 0 (0-15.7) | 14.5  (15.4) | 13.4 (0-20.8) | 0.263 |
| **Angiopoietin-2** | 14.3  (10.3) | 14.5 (5.2-20.1) | 8 (7.9) | 10.7 (0-13.6) | 8.5 (13.2) | 0 (0-14.8) | 11.1  (13.6) | 11.4 (0-14.7) | 0.103 |
| **Angiostatin/Plasminogen** | 0.6 (2.7) | 0 (0-0) | 0 (0) | 0 (0-0) | 0.7 (3.1) | 0 (0-0) | 0 (0) | 0 (0-0) | 0.567 |
| **Amphiregulin** | 0 (0) | 0 (0-0) | 0 (0) | 0 (0-0) | 0 (0) | 0 (0-0) | 0 (0) | 0 (0-0) | N/A |
| **Artemin** | 0.6 (2.7) | 0 (0-0) | 0 (0) | 0 (0-0) | 0.5 (2.3) | 0 (0-0) | 1.2 (3.6) | 0 (0-0) | 0.546 |
| **Coagulation Factor III** | 0 (0) | 0 (0-0) | 0 (0) | 0 (0-0) | 0 (0) | 0 (0-0) | 0 (0) | 0 (0-0) | N/A |
| **CXCL 16** | 3.3 (7.2) | 0 (0-0) | 1.2 (3.7) | 0 (0-0) | 0.5 (2.4) | 0 (0-0) | 1.2 (3.6) | 0 (0-0) | 0.427 |
| **DPPIV** | 41.5  (34.2) | 38 (15.1-66.6) | 38.9 (29) | 46.2 (7.9-60.8) | 25.1  (22.5) | 22.9 (5.6-33.5) | 43 (25.6) | 50.3 (26.3-  59.1) | 0.197 |
| **EGF** | 0 (0) | 0 (0-0) | 0 (0) | 0 (0-0) | 0 (0) | 0 (0-0) | 0 (0) | 0 (0-0) | N/A |
| **EG-VEGF** | 0.5 (2.3) | 0 (0-0) | 2.5 (5.3) | 0 (0-0) | 0 (0) | 0 (0-0) | 0.6 (2.7) | 0 (0-0) | 0.085 |
| **Endoglin** | 0 (0) | 0 (0-0) | 0 (0) | 0 (0-0) | 0 (0) | 0 (0-0) | 0 (0) | 0 (0-0) | N/A |
| **Endostatin/Collagen**  **XVIII** | 21.5  (16.8) | 20.5 (6.2-32) | 17.6  (14.6) | 21.4 (0-27.5) | 19 (17.5) | 18.5 (0-33.8) | 22 (17.3) | 21.3 (6.3-36) | 0.922 |
| **Endothelin-1** | 12.1  (10.3) | 10.8 (5-16.5) | 5.3 (7.9) | 0 (0-11.2) | 7.4 (12.9) | 0 (0-12.4) | 11.6  (15.2) | 5.9 (0-19.6) | 0.129 |
| **FGF acidic** | 0 (0) | 0 (0-0) | 0 (0) | 0 (0-0) | 0 (0) | 0 (0-0) | 0 (0) | 0 (0-0) | N/A |
| **FGF basic** | 0 (0) | 0 (0-0) | 0 (0) | 0 (0-0) | 0 (0) | 0 (0-0) | 0.8 (3.4) | 0 (0-0) | 0.392 |
| **FGF-4** | 0 (0) | 0 (0-0) | 0 (0) | 0 (0-0) | 0 (0) | 0 (0-0) | 0 (0) | 0 (0-0) | N/A |
| **FGF-7** | 0 (0) | 0 (0-0) | 0 (0) | 0 (0-0) | 0 (0) | 0 (0-0) | 0 (0) | 0 (0-0) | N/A |
| **GDNF** | 0 (0) | 0 (0-0) | 0 (0) | 0 (0-0) | 0 (0) | 0 (0-0) | 0 (0) | 0 (0-0) | N/A |
| **GM-CSF** | 0 (0) | 0 (0-0) | 0 (0) | 0 (0-0) | 0 (0) | 0 (0-0) | 0 (0) | 0 (0-0) | N/A |
| **HB-EGF** | 0.6 (2.6) | 0 (0-0) | 1.9 (4.8) | 0 (0-0) | 0 (0) | 0 (0-0) | 1.6 (4.9) | 0 (0-0) | 0.304 |
| **HGF** | 0 (0) | 0 (0-0) | 0 (0) | 0 (0-0) | 0 (0) | 0 (0-0) | 0 (0) | 0 (0-0) | N/A |
| **IGFBP-1** | 19.7  (23.2) | 11.9 (0-34.4) | 30.9  (27.1) | 26.1 (7.6-49.9) | 19.5  (25.8) | 13.4 (0-20.1) | 27.1  (29.8) | 19.4 (12-38.7) | 0.217 |
| **IGFBP-2** | 17.4  (17.3) | 15 (0-22.8) | 20.6  (19.7) | 21.6 (0-36.3) | 24.3  (24.3) | 19.8 (5.1-34.8) | 32.9 (27.7) | 27.6 (5.3-58.2) | 0.326 |
| **IGFBP-3** | 75.1 (31) | 80.6 (61.1-92.6) | 57.3 (29) | 60.6 (40.9-77) | 62 (46.5) | 65.9 (19.8-  97.9) | 65.1 (37) | 68 (37.6-88) | 0.300 |
| **IL-1β** | 0.5 (2.3) | 0 (0-0) | 0 (0) | 0 (0-0) | 0 (0) | 0 (0-0) | 0.6 (2.6) | 0 (0-0) | 0.567 |
| **IL-8** | 0 (0) | 0 (0-0) | 0 (0) | 0 (0-0) | 0 (0) | 0 (0-0) | 0 (0) | 0 (0-0) | N/A |
| **LAP (TGF-β1)** | 3.3 (6.1) | 0 (0-5.1) | 2.9 (5.3) | 0 (0-5) | 0 (0) | 0 (0-0) | 0.6 (2.6) | 0 (0-0) | **0.037 #** |
| **Leptin** | 14.5  (21.9) | 0 (0-26.8) | 7.9 (13.6) | 0 (0-13.1) | 1.7 (5.2) | 0 (0-0) | 5.3 (18.3) | 0 (0-0) | 0.052 |
| **MCP-1** | 0 (0) | 0 (0-0) | 0 (0) | 0 (0-0) | 0 (0) | 0 (0-0) | 0 (0) | 0 (0-0) | N/A |
| **MIP-1a** | 0.6 (2.8) | 0 (0-0) | 0 (0) | 0 (0-0) | 0 (0) | 0 (0-0) | 1.4 (6.1) | 0 (0-0) | 0.567 |
| **MMP-8** | 1.4 (4.6) | 0 (0-0) | 2.2 (5.6) | 0 (0-0) | 1.2 (3.7) | 0 (0-0) | 3.4 (10.6) | 0 (0-0) | 0.904 |
| **MMP-9** | 27.6 (26) | 19.4 (14.1-40.5) | 15.7  (15.5) | 15.2 (0-22.7) | 17.7  (19.1) | 13.4 (0-29.6) | 27.3  (33.3) | 17.6 (0-36.6) | 0.377 |

| **NRG1-β1** | 0 (0) | 0 (0-0) | 0 (0) | 0 (0-0) | 0 (0) | 0 (0-0) | 0 (0) | 0 (0-0) | N/A |
| --- | --- | --- | --- | --- | --- | --- | --- | --- | --- |
| **Pentraxin 3 (PTX3)** | 9.6 (8.1) | 11 (0-14.8) | 7.9 (8.8) | 5.9 (0-14) | 6 (8.7) | 0 (0-14.4) | 9.9 (12.9) | 0 (0-17.7) | 0.681 |
| **PD-ECGF** | 2.8 (5) | 0 (0-5.1) | 6.3 (8.2) | 0 (0-15.9) | 0.6 (2.8) | 0 (0-0) | 4.2 (5.9) | 0 (0-10.9) | **0.037 *** |
| **PDGF-AA** | 3.2 (5.9) | 0 (0-5.2) | 2.9 (5.4) | 0 (0-5.2) | 0 (0) | 0 (0-0) | 2.1 (5.4) | 0 (0-0) | 0.119 |
| **PDGF-AB/PDGF-BB** | 0 (0) | 0 (0-0) | 0 (0) | 0 (0-0) | 0 (0) | 0 (0-0) | 0 (0) | 0 (0-0) | N/A |
| **Persephin** | 0.6 (2.6) | 0 (0-0) | 2 (4.8) | 0 (0-0) | 1.6 (3.9) | 0 (0-0) | 2.4 (5.1) | 0 (0-0) | 0.576 |
| **Platelet Factor 4 (PF4)** | 122.3  (30.9) | 113.8 (104.7-  131.3) | 92.8  (26.1) | 95.4 (85.5-  110.4) | 79 (50.3) | 82 (33.5-116) | 90.7  (41.8) | 94.2 (65.5-  119.5) | **0.006 #!** |
| **PIGF** | 0 (0) | 0 (0-0) | 0 (0) | 0 (0-0) | 0 (0) | 0 (0-0) | 0 (0) | 0 (0-0) | N/A |
| **Prolactin** | 1.5 (5) | 0 (0-0) | 0.6 (2.6) | 0 (0-0) | 0.6 (2.8) | 0 (0-0) | 0 (0) | 0 (0-0) | 0.556 |
| **Serpin B5** | 0 (0) | 0 (0-0) | 0 (0) | 0 (0-0) | 0 (0) | 0 (0-0) | 0.8 (3.4) | 0 (0-0) | 0.392 |
| **Serpin E1** | 92.2  (47.3) | 85.3 (67.4-97.7) | 71 (29.5) | 71.6 (52.3-88.6) | 62.2  (41.4) | 49.1 (39.1-  79.8) | 50.9  (28.3) | 44.9 (30.7-  57.7) | **0.001 # ƚ** |
| **Serpin F1** | 108.2  (30.5) | 97.1 (82.5-  131.9) | 84.2  (25.7) | 78.2 (71.7-  102.1) | 92.6 (40) | 94.3 (67.5-  114.2) | 93.3 (34) | 85.7 (67-  115.8) | 0.121 |
| **TIMP-1** | 78.2  (46.2) | 66.7 (48.5-95.5) | 67.5 (44) | 64.7 (40.7-77.6) | 91.5  (38.8) | 88.3 (64.4-  113.2) | 81.7  (34.2) | 74.1 (67.5-  100.9) | 0.092 |
| **TIMP-4** | 4 (7.3) | 0 (0-6.4) | 4.1 (6.6) | 0 (0-11.2) | 0.5 (2.3) | 0 (0-0) | 2 (6.6) | 0 (0-0) | 0.119 |
| **Thrombospondin-1** | 129.1  (49.6) | 122.3 (95.2-  142) | 97.5  (29.7) | 99.8 (85.7-120) | 80.7  (62.8) | 78.5 (30.9-  102.3) | 94.7  (43.2) | 85 (71.3-  133.5) | **0.005 #** |
| **Thrombospondin-2** | 4.2 (6.3) | 0 (0-10.3) | 1.8 (4.6) | 0 (0-0) | 2.5 (6.2) | 0 (0-0) | 5.2 (10.3) | 0 (0-10.3) | 0.412 |
| **uPA** | 0.6 (2.6) | 0 (0-0) | 0 (0) | 0 (0-0) | 0.6 (2.5) | 0 (0-0) | 0 (0) | 0 (0-0) | 0.567 |
| **Vasohibin** | 0 (0) | 0 (0-0) | 0 (0) | 0 (0-0) | 0 (0) | 0 (0-0) | 1 (4.3) | 0 (0-0) | 0.392 |
| **VEGF**  **VEGF-C** | 1.8 (4.3) | 0 (0-0) | 1.8 (4.4) | 0 (0-0) | 0 (0) | 0 (0-0) | 2.5 (5.2) | 0 (0-0) | 0.255 |
|  | 0 (0) | 0 (0-0) | 0 (0) | 0 (0-0) | 0 (0) | 0 (0-0) | 1.2 (5.2) | 0 (0-0) | 0.392 |
